# Obstructed right Duari hernia

**DOI:** 10.4314/gmj.v55i3.9

**Published:** 2021-09

**Authors:** Ugochukwu U Nnadozie, Otuu Onyeyirichi, Charles C Maduba, Andrew C Ekwesianya

**Affiliations:** 1 Division of Plastic Surgery, Department of Surgery, Alex Ekwueme Federal University Teaching Hospital Abakaliki, Ebonyi State, Nigeria; 2 Department of Surgery, Ebonyi State University, Abakaliki, Ebonyi State, Nigeria; 3 General Surgery Unit, Department of Surgery, Alex Ekwueme Federal University Teaching Hospital Abakaliki, Ebonyi State Nigeria

**Keywords:** Duari hernia, groin hernia, inguinal hernia, obstructed femoral hernia, strangulated hernia

## Abstract

The caecum and appendix are uncommon contents of femoral hernia (Duari hernia). Diagnosis is usually intraoperative. We report a rare case of obstructed right femoral hernia in a 65-year-old woman. She was admitted into the accident and emergency department because of sudden irreducibility of a previously reducible right groin swelling of 5 years duration. She had obstructive symptoms with an irreducible right groin mass clinically diagnosed as obstructed right femoral hernia. A combination of infra-inguinal transverse incision and a lower midline laparotomy incision was used. The intraoperative findings included the herniation of the caecum and appendix into the right femoral canal. Patient had an uneventful recovery. Duari hernia is uncommon. A high index of suspicion and an experienced surgeon, who can handle uncommon findings should be involved in the management of obstructed femoral hernias.

**Funding:**

None declared

## Introduction

Femoral hernias account for around 2.8%–4% of all groin hernias, are less common than inguinal hernias and occur more frequently in females.^1^,^2^ Anatomically, they represent herniations of the peritoneal sac through the femoral ring into the femoral canal, lying postero-inferiorly to the inguinal ligament. The femoral canal is bounded by the femoral vein laterally, the inguinal ligament anteriorly, the pectineal ligament posteriorly and the lacunar ligament medially.^3^

Abdominal hernia sac commonly consists of omentum or small bowel, but uncommon contents like caecum, appendix, colon, Meckel's diverticulum, ovaries, testes, stomach and kidneys have been reported. 4–6. When a femoral hernia contains caecum and appendix it is called Duari hernia after the person (Duari), who first reported it. 6 When compared to inguinal hernia, femoral hernias present more often with incarceration and thus have substantial risk of emergency operations with higher rates of bowel resection, complications, and mortality. 1

We present a case of obstructed right femoral hernia containing caecum and appendix diagnosed incidentally at operation, highlighting the need for skilled surgeon to be involved in the management of femoral hernia especially if there is obstruction.

## Case Report

The patient gave her consent for the use of her information and pictures for publication. Ethical approval was gotten from the Research and Ethics Committee (REC) of Alex Ekwueme Federal University Teaching Hospital Abakaliki (REC APPROVAL NUMER 15/05/2020 – 18/05/2020)

**Figure 1:**
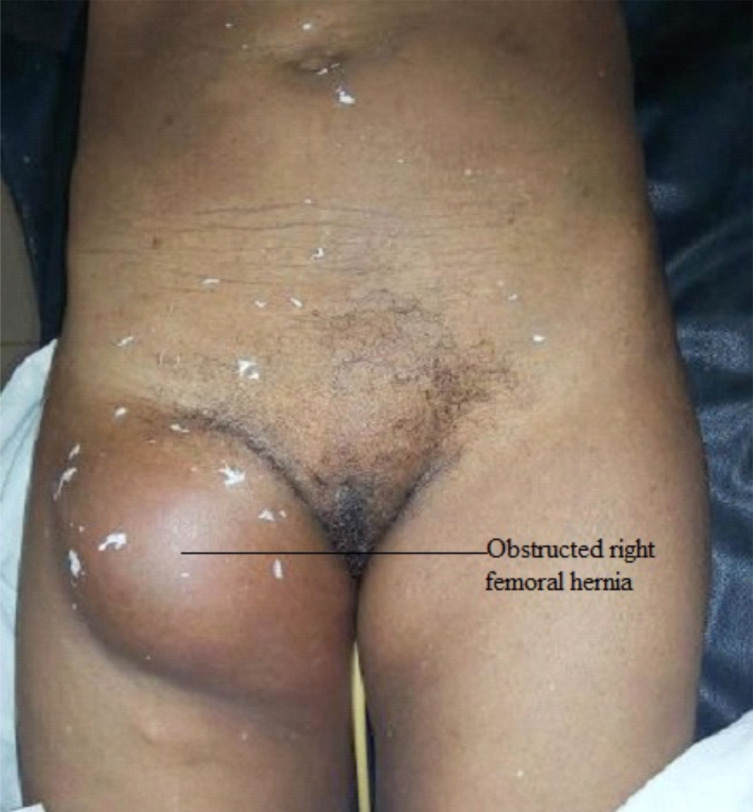
Obstructed right femoral hernia at presentation

65-year-old woman was referred to our accident and emergency room from a primary health centre with a previously reducible right groin swelling of 5 years that became suddenly irreducible and painful five days before presentation. There were associated colicky lower abdominal pain, bilious vomiting, and constipation. She had no significant medical comorbidities. On assessment, the patient was in mild painful distress with tachypnea otherwise she was stable. There was a tender 10x10x7cm mass on the right groin below the groin crease and infero-lateral to the pubic tubercle (Figure 1).

The mass extended to the medial aspect of right upper thigh with overlying erythema and an absent cough impulse. There were hyperactive bowel sounds and empty rectum on digital rectal examination. Her full blood count was within normal limits. She had hypokalemia of 3.2mmol/l.

**Figure 2:**
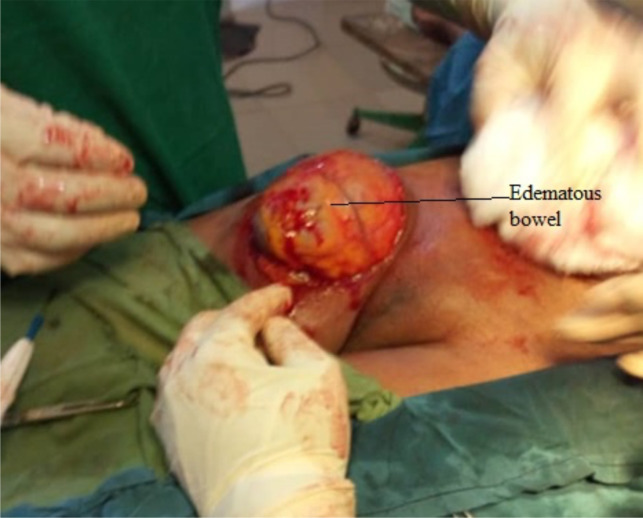
Intraoperative finding femoral hernia; irreducible via infra-inguinal incision

Abdominal ultrasound scan demonstrated a heterogeneous mass lateral to the femoral vessels that appeared to be arising from the abdominal cavity through a defect of 10.1mm in diameter. The mass measured 4.6cm in diameter and contained bowel loops with reduced vascular flow on doppler. The bowel loops had increased intraluminal fluid and were surrounded by echo-rich fluid. A diagnosis of obstructed right femoral hernia to rule out strangulation was made. Patient was resuscitated with intravenous fluids, placed on antibiotics, and had electrolyte abnormality corrected. She was prepared and booked for emergency operation to relieve the obstruction in the femoral hernia by the consultant surgeon. The mass was approached through an infra-inguinal transverse incision (Figure 2).

**Figure 3:**
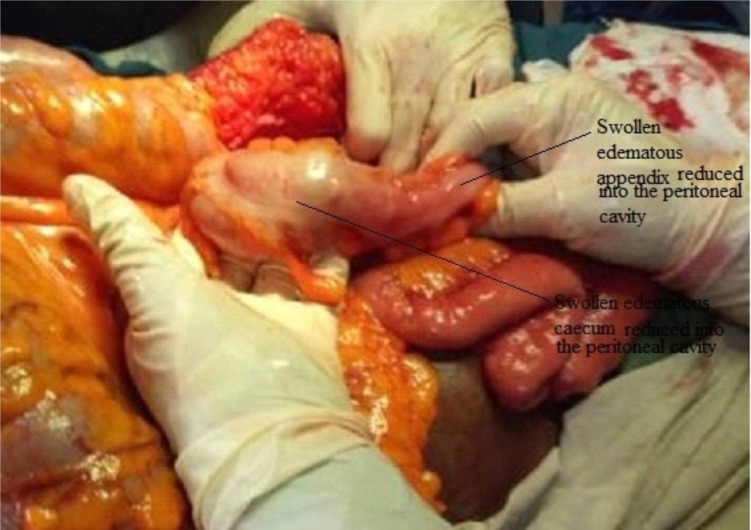
Edematous caecum and appendix reduced into the peritoneal cavity through laparotomy

The sac was carefully dissected out. It contained strawcolored fluid together with an edematous but viable caecum and appendix. A lower midline incision was made to gain access to the peritoneal cavity with the demonstration of the whole caecum herniating through defect in the femoral ring. The inguinal ligament was divided from below and the content of the sac was reduced (Figure 3). The inguinal ligament was repaired with nylon 3/0 suture in three strings and the femoral defect was repaired by successfully approximating the pectineal and inguinal ligaments using three interrupted nylon 2 sutures. The patient had uneventful postoperative recovery on fluids, antibiotics and analgesics and vitamin supplements. She was discharged on the fifth post-operative day.

She was followed up in the clinic for six months, without wound infection or recurrence.

## Discussion

This is a case of obstructed Duari hernia in an elderly woman. Failure of mesenteric fixation with abnormal mobility of the caecum and ascending colon migrates the caecum into the femoral canal during development as seen in 10–20% of the population has been said to lead to femoral hernia.^7^

The common clinical features of abdominal pain, painful groin swelling, erythema of the groin and sudden irreducibility of a long-standing groin swelling with a risk factor of elderly female gender were seen in our case as noted in other studies. 1, 2, 8 Strangulation is imminent in femoral hernias because of the narrow femoral ring as was seen in this index case where the caecum was already dilated and edematous and trapped by a tightly constricted neck. A case of strangulated femoral hernia was reported to have even masqueraded as groin abscess.^9^

Clinical examination is of limited value in identifying the contents of the femoral hernia. 10 The preoperative ultrasound scan interpretation of this case was correct about the presence of bowel loops but was non-specific about the parts of the herniating loop. Multidetector computed tomography (CT) with multiplanar reconstructs and femoral vein compression sign on a CT scan will be useful in differentiating a femoral from an inguinal hernia and also identify the hernia contents.^8^ However, even though CT was not available in our center, we do not think it would have contributed much to the clinical assessment of the emergency scenario with urgent need for exploration and intervention. Our findings in this case, like in most hernias with uncommon contents was incidental at operation^5^,^6^ Surgical options for femoral hernia include, closed (laparoscopy) and the classical open approaches which include Lockwood's infra-inguinal, Lotheissen's trans-inguinal, and McEvedy's high incisions.^8^,^10^, We combined infra-inguinal incision with a lower midline laparotomy incision. Through the infra-inguinal incision, we were able to suck out the toxic fluid content of the sac obviating possible sepsis and the content were demonstrated. We also incised the inguinal ligament to free the narrow neck. The lower midline incision aided us to reduce the edematous caecum and appendix gently and safely into the peritoneal cavity. It also helped ruled out other incidental abdominal findings like gangrenous bowel. This approach has also been described in other reports,^4^,^9^ and we found it useful in this emergency situation where speed, accuracy and skill is of essence. McEvedy's high incision gives similar access, with added advantage of single incision but was not the surgeon's choice. Our use of antibiotics may have contributed to smooth postoperative condition devoid of sepsis and wound infection.

## Conclusion

Duari hernia is uncommon and, preoperative diagnosis remains a difficult challenge. There is need for high index of suspicion, and an experienced surgeon who can handle uncommon findings should be involved in management of obstructed femoral hernias.

## References

[1] Dahlstrand U, Wollert S, Nordin P, Sandblom G, Gunnarsson U (2009). Emergency femoral hernia repair: a study based on a national register. Ann Surg.

[2] Bay-Nielsen M, Kehlet H, Strand L, Malmstrøm J, Andersen FH, Wara P, Juul P, Callesen T (2001). Quality assessment of 26,304 herniorrhaphies in Denmark: a prospective nationwide study. Lancet.

[3] Sinnatamby CS (2011). Last's anatomy: regional and applied.

[4] Patel RB, Vasava N, Hukkeri S (2012). Non-obstructed femoral hernia containing ascending colon, caecum, appendix and small bowel with concurrent bilateral recurrent inguinal hernia. Hernia.

[5] D Kidmas AT, Iya D, Yilkudi GM, Nnadozie U (2004). Acute appendicitis in inguinal hernia: Report of two cases. East Afr Med J.

[6] Duari M (1966). Strangulated femoral hernia—a Richter's type containing caecum and base of appendix. Postgrad Med J.

[7] Rogers RL, Harford FJ (1984). Mobile cecum syndrome. Dis Colon Rectum.

[8] Goh IY, Sandstrom AL, Stapleton T, Aseervatham R, Grieve DA (2017). The Duari hernia and recognition of the femoral vein compression sign. BMJ Case Rep.

[9] Arkoulis N, Savanis G, Simatos G (2012). Richter's type strangulated femoral hernia containing caecum and appendix masquerading as a groin abscess. J. Surg. Case Rep.

[10] Jin z, Imtiaz MR, Nnajiuba H, Samlalsingh S, Ojo A (2016). De Garengeot's Hernia: Two Case Reports with Correct Preoperative Identification of the Vermiform Appendix in the Hernia. Case Rep Surg.

